# 

**Published:** 1868-06

**Authors:** 


### EDITORIAL DEPARTMENT.

*The American Dental Association.* The next meeting of this Association will be held at Niagara Falls on the last Tuesday of July, and if it be not considered presumptuous to offer a little advice to the officers of so august an assemblage, we should recommend courtesy and politeness to all regular delegates when presenting their credentials and endeavoring to express their opinion on the questions under debate.

On entering the meeting of the Association, held last summer at Hopkins Hall, Cincinnati, we were forcibly reminded of the arbitrary manner of the president of a "court martial" (to which it was once our ill-fortune to be summoned as a witness,) towards the advocates engaged in the case on trial.

As it was our first visit to a meeting of the American Dental Association, we were unacquainted with the manner in which its business was conducted, and supposed that at least delegates who had grown gray in the profession and had its advancement greatly at heart, would be treated as gentlemen, and attentively listened to when they attempted to address the chair.

We had been in the hall but a short time before this supposition on our part was wholly obliterated; but consoled ourself with the thought that as we had determined to be a silent observer on this our first visit, no such rebuffs would reach us. On recovering somewhat from our surprise, the necessity existing for a visit to the Treasurer of the Association, who occupied a desk in the rear of the audience, occurred to us.

Before announcing ourself and while awaiting our turn to reach the desk, we found that, instead of a kind reception in a strange place, the delegates were treated in the same manner by the Treasurer, as the Presiding officer had been treating the old members of the profession on the floor, and for a few moments we hesitated to submit ourself to the same treatment.

But, curiosity, as to how matters were conducted in such an assemblage, prevailed, and we passed through the ordeal, signing our name and paying the annual fee.

In concluding, we shall take the liberty of suggesting to the Association that if they are unacquainted with the dispositions of the future candidates for the offices in their gift, a committee of inquiry be appointed, so that it may be a pleasure, instead of trial, to attend a meeting of the American Dental Association, and participate in its proceedings.

*Time of Meeting of American Dental Association.*—The eighth session of this Association will be held in Grant's Hall, Niagara Falls, beginning on Tuesday, July 28th 1868.

The following arrangements have been made with regard to accommodations. The International Hotel will receive members of the Association at $4.00 per day, a reduction of fifty cents per day from their regular terms. The Spencer House charges $3.50 per day. By giving timely notice to the committee of arrangements, apartments will be reserved for members of the Association, especially those accompanied by their families, at either of these hotels.

Geo. B. Snow,<br>B. T. Whitney,<br>A. P. Southwick, } Committee of Arrangements.

### TO [illegible]DERS AND CORRESPONDENTS.

Communications intended for publication, and exchange journals and papers, should be addressed to the EDITORS OF THE AMERICAN JOURNAL OF DENTAL SCIENCE, Baltimore, Md. All letters relating to the business of the journal, or containing cash remittances, to be directed to Messrs. SNOWDEN & COWMAN. Articles for publication should be received by the editors at least three weeks previous to the first day of the month on which the number in which it is designed for them to appear, is issued.

☞ The American Journal of Dental Science will contain fifty pages of reading matter in each number, making a volume of not less than *Six Hundred* pages;

**EXCLUSIVE OF ADVERTISEMENTS.**

